# Spatio-temporal pattern recognizers using spiking neurons and spike-timing-dependent plasticity

**DOI:** 10.3389/fncom.2012.00084

**Published:** 2012-10-10

**Authors:** James Humble, Susan Denham, Thomas Wennekers

**Affiliations:** Centre for Robotic and Neural Systems, Cognition Institute, Plymouth UniversityPlymouth, UK

**Keywords:** sequence learning, synfire chains, spiking neurons, spike-timing-dependent plasticity, neural automata

## Abstract

It has previously been shown that by using spike-timing-dependent plasticity (STDP), neurons can adapt to the beginning of a repeating spatio-temporal firing pattern in their input. In the present work, we demonstrate that this mechanism can be extended to train recognizers for longer spatio-temporal input signals. Using a number of neurons that are mutually connected by plastic synapses and subject to a global winner-takes-all mechanism, chains of neurons can form where each neuron is selective to a different segment of a repeating input pattern, and the neurons are feed-forwardly connected in such a way that both the correct input segment and the firing of the previous neurons are required in order to activate the next neuron in the chain. This is akin to a simple class of finite state automata. We show that nearest-neighbor STDP (where only the pre-synaptic spike most recent to a post-synaptic one is considered) leads to “nearest-neighbor” chains where connections only form between subsequent states in a chain (similar to classic “synfire chains”). In contrast, “all-to-all spike-timing-dependent plasticity” (where all pre- and post-synaptic spike pairs matter) leads to multiple connections that can span several temporal stages in the chain; these connections respect the temporal order of the neurons. It is also demonstrated that previously learnt individual chains can be “stitched together” by repeatedly presenting them in a fixed order. This way longer sequence recognizers can be formed, and potentially also nested structures. Robustness of recognition with respect to speed variations in the input patterns is shown to depend on rise-times of post-synaptic potentials and the membrane noise. It is argued that the memory capacity of the model is high, but could theoretically be increased using sparse codes.

## 1. Introduction

In 1982, when trying to account for observed precise sequences of neural firing with long inter-spike delays, Abeles coined the term “synfire chain” (Abeles, 1982, 1991). Some delays seen between spikes of simultaneously recorded neurons were extremely long, but repeated with a precision in the millisecond range. This was difficult to understand given that the firing patterns of single neurons look very noisy and can often be well described as rate-modulated Poisson processes. A possible mechanism to produce these delays was the postulated synfire chain consisting of feed-forwardly connected populations of cells. Each population would contain neurons with excitatory “diverging-converging” connections to neurons in the next population. The populations are defined by their connectivity and therefore symbolize the order of activation; individual neurons may take part in more than one (and in fact many) populations. Activity propagates from population to population in a synchronous manner, which can be shown to be a stable and precise process when the compound post-synaptic potential caused by neurons in one population on the their targets in the next population is significantly larger than the noise level (Abeles, 1991; Wennekers and Palm, 1996; Wennekers, 2000). As activity can travel through a synfire chain in the same manner many times, the delay between two neurons along the chain is fixed as observed originally in the experimental recordings. Abeles' synfire chain model has homogeneous axonal delays between populations, an assumption that can be relaxed to include an axonal delay distribution. Connections may then straddle intermediate populations. Such a set-up was called a “synfire braid” by Bienenstock (1995). Izhikevich (2006) has shown that similar connectivity and activation patterns (called “polychronous waves”) can be generated in networks of spiking neurons as a result of random excitation and a learning rule that depends on pre- and post-synaptic spike-times [“spike-timing-dependent plasticity,” (STDP), cf., Bi and Poo, 1998; Morrison et al., 2008].

Experimental support for synfire chains is difficult to obtain as one needs to simultaneously record from many neurons at once in order to find correlated pairs. Current techniques are limited to record from up to a few 100 sparsely located neurons and are at best able to record a few neurons from a chain. Nevertheless, precisely repeating firing patterns have been observed (Prut et al., 1998; Nadasdy et al., 1999; Ikegaya et al., 2004). For example, Ikegaya et al. (2004) describe precise repetitions of spontaneous patterns in neocortical neurons *in vivo* and *in vitro* and Nadasdy et al. (1999) found repeating spike sequences in awake and asleep rat hippocampus. It has further been shown that sequences can be related to a monkey's behavior (Prut et al., 1998; Shmiel et al., 2005, 2006). Prut et al. (1998) observed both (1) precise firing sequences in cortical activity which spanned hundreds of milliseconds and (2) their correlation with animal behavior in one study. Hahnloser et al. (2002) also reported repeating spike-patterns in the song-system of birds that are clearly related to syllable generation. More recently, Ayzenshtat et al. (2010) described spatio-temporal patterns within and across early visual areas that contained enough information to reliably discriminate between stimulus categories. Some of these findings, however, have been challenged. For example, Mokeichev et al. (2007) re-examined the observation of repeating spontaneous patterns and suggest that they may not be significant but appear by chance. Furthermore, even though the precisely repeating firing patterns could be the result of synfire chains, these patterns do not ultimately prove the existence of synfire chains.

A question following these ideas is “how can synfire chains form?” Early attempts to derive them in neural networks subject to some kind of self-organization have been moderately successful. Bienenstock and Doursat (1991), for example, reported that exciting neurons synchronously in a random network could lead to the formation of synfire chains. Another study by Prugel-Bennett and Hertz (1996) also attempted to learn synfire chains in random networks but found that closed loops formed. These loops are a problem as once activity enters a loop it never exits again; consequently, activity may never propagate fully through a synfire chain. In these studies, the total synaptic strength for a neuron's efferent synapses was limited; if this was not the case, connections would strengthen uncontrollably. A more recent study by Izhikevich (2006) used STDP in spiking neuron networks subject to unspecific random excitation. In these very large-scale simulations complex recurrently connected web-like structures of specific synaptic pathways formed that carried repeating “polychronous waves” with many properties of synfire chains, but a broad distribution of possible delays between neurons. In the more abstract set-up of Markov chains, Ay and Wennekers (2003) also demonstrated the formation of web-like nested transition structures in the joint state space of *N* units using timing-dependent plasticity. These transition structures could be linked to finite state automata (Wennekers and Ay, 2005) which suggests that they could be employed for neural state-based computations. An alternative hypothesis for computing with polychronous waves has been suggested by Izhikevich (2009).

In the present study we took a different approach to learn synfire chains assuming that they may not form autonomously in a network, but rather by spatio-temporal driving activity which may come from other areas or the sensory surfaces. Repeated inputs could cause their target neurons to learn a specific firing pattern. Essentially, we attempted to recognize a repeated spatio-temporal pattern with a sequence of neurons. When successful, several neurons respond one after the other, each recognizing a segment of the input pattern. The learning of short repeated spatio-temporal patterns has been studied and analysed previously (Masquelier et al., 2008, 2009; Humble et al., 2011). It has been shown that a neuron with STDP is able to learn and respond to the beginning of a repeated spatio-temporal pattern even when it is embedded in a statistically identical carrier signal. Furthermore, Masquelier et al. (2009) reported that if, instead of one neuron, several neurons are competing for the ability to respond and learn the pattern, they each learn a segment of the pattern. When inspecting the whole learnt pattern, however, a limitation becomes apparent: each neuron is responding to a unique learnt segment of the input regardless of when it appears. Therefore one could take an input pattern, switch the segments around, and the same neurons in the trained network would fire, albeit in a different order. We therefore were interested in whether it was possible to learn the order of the responses. If so, the network would learn to recognize a spatio-temporal input pattern in such a way that a chain of activity in the trained network would only propagate if a specific spatio-temporal input pattern supported it from the beginning to its end.

To learn temporal order, it seems natural to introduce plastic lateral connections which reinforce and prime a subsequent neuron's firing. A neuron will then not only rely on the input pattern but also on previously responding neurons. We found that by including lateral connections with STDP the model could learn the order of neural firing activity and it was possible to have an “accepting neuron” that only responded if a learnt pattern was presented fully, thereby signaling when the whole pattern was contained in the input stream.

Such a set-up is analogous to finite state automata where an accepting state is reached only if previous states are transitioned correctly. The currently firing neurons represent “the state” of a recognizer for the input pattern and the momentary inputs act as “symbols” that drive the recognizer through its states. If at the end of the input the final neurons of the synfire chain fire, this signifies that the sequence of input symbols has been recognized correctly. This is functionally a simple finite state automaton with no loops in the transition graph. In Wennekers and Palm (2007) and Wennekers (2006), we have argued how more complex neural systems of this sort can be systematically constructed.

In the present work, we take a step toward algorithms that can learn spiking neuron automata with accepting states/neurons. We present a learning scenario whereby a biologically based learning rule learns temporal sequences comprised of several neurons firing one after another. Furthermore, we find that by using plastic lateral connections our network's neurons can learn to respond only when temporally appropriate. This differs from previous work such as that of Masquelier et al. (2009) because we include plastic lateral connections.

The paper is organized as follows. The next section introduces the neuron and network model used, as well as the learning rule and training paradigm. Section 3 presents the main results split into several subsections. At first, proof-of-concept simulations for sequence learning and recognition are given followed by some analyses of parameter dependences. It is then shown that previously learnt chains can be sequenced into longer chains. It is argued that learnt chains contain a high amount of information and a certain robustness against variations in the speed of stimuli. Factors that limit robustness and memory capacities are discussed. The discussion section finally puts the results into a wider context and outlines their relevance for state-based neural computing architectures.

## 2. Materials and methods

**Figure 1:**
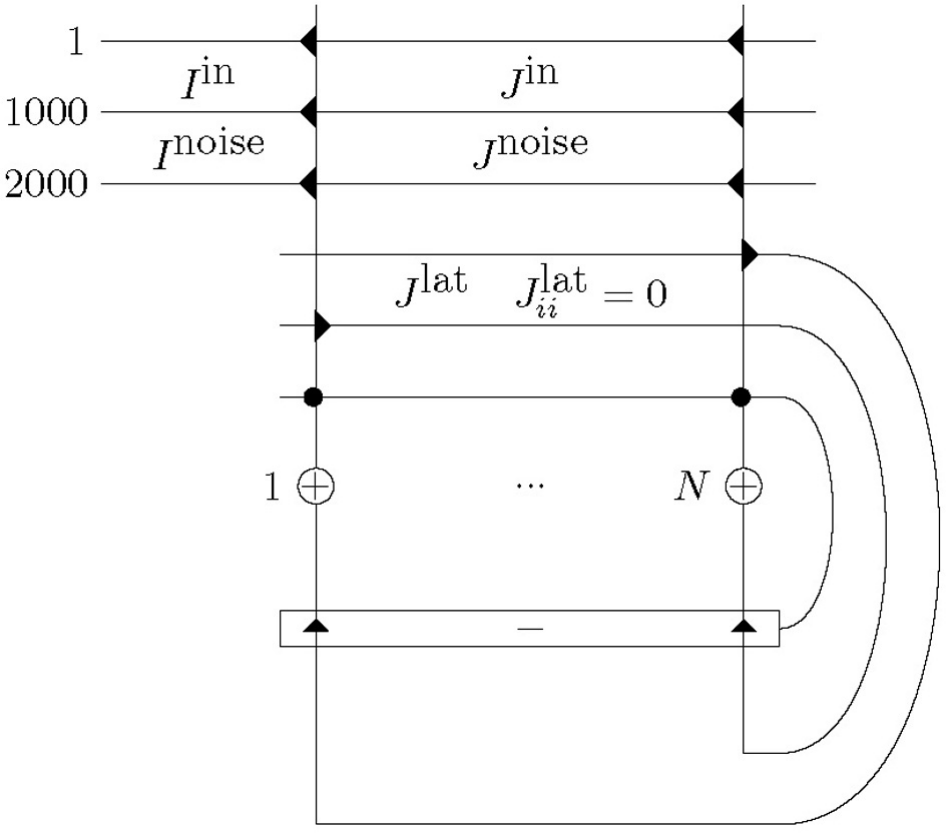
**Network structure with *N* excitatory neurons (+) and one inhibitory neuron (−), and corresponding excitatory (triangle) and inhibitory (circle) synapses.** Excitatory neurons are connected to one another with plastic mutual connections, *J*^lat^. They are in competition with each other via one inhibitory neuron. Each excitatory neuron receives input consisting of a total of *N*_input_ = 2000 afferents. Half of these, *I*^in^, alternate between presenting a repeated spatio-temporal pattern (for 50 ms) and Poisson input (for at least 50 ms). The other half, *I*^noise^, continuously present Poisson trains. This input is presented to each of the excitatory neurons, however, across the excitatory neurons the input has only the repeated spatio-temporal pattern in common as the alternating and continuous Poisson trains are different. Furthermore, the repeated spatio-temporal pattern is presented to each neuron at the same time. All input synapses, *J*^in^ and *J*^noise^, and lateral synapses, *J*^lat^ (except *J*^lat^_*ii*_), are plastic and *J*^lat^_*ii*_ = 0.

Our network comprised *N* = 20 excitatory neurons with one inhibitory neuron providing winner-takes-all competition: see Figure 1. The size of *N* can be small, if only short sequences are to be learned. The excitatory neurons were laterally connected to all other excitatory neurons with synapses that were plastic throughout a simulation.

Input, *I*, consisted of 2000 afferents to each excitatory cell. One half of the afferents continuously transmitted independent Poisson spike trains, *I*^noise^. The other half alternated between independent Poisson spike trains and a fixed spatio-temporal spike-pattern of 50 ms duration inserted at random times, *I*^in^ (longer patterns are possible and do not change the results much). These 50 ms-patterns were generated by Poisson processes as well, but the patterns were fixed for the duration of the simulations and identically applied to all excitatory neurons. The rate of the Poisson spike trains and the repeated spatio-temporal pattern were 54 Hz. Additionally, 10 Hz Poisson noise was added to all afferents including during the presentation of the frozen patterns. The input synapses, *J*^in^ and *J*^noise^, and lateral synapses, *J*^lat^, are plastic for the duration of the simulation. This set-up follows Masquelier et al. (2008, 2009) and provides a scenario where a pattern is embedded in a statistically identical carrier signal. It differs from Masquelier et al.'s work only by the inclusion of trainable recurrent connections.

All neurons were leaky-integrate-and-fire cells, Equation (1), with synapses described by alpha functions, Equation (2). τ_*m*_ = 10 ms is the membrane time constant, τ_*r*_ = 1 ms is the rise-time constant of the alpha synapse and τ_*f*_ = 5 ms the decay constant. A firing threshold of θ = 1 was used. The inhibitory neuron was an integrate and fire neuron with the same parameters as the excitatory neurons. For convenience, inhibitory synapses had the same dynamics than excitatory ones, but were non-plastic. Likewise, excitatory synapses on the inhibitory cell did not learn. Their values were all identical and set such that a spike in the excitatory network reliably triggered activity in the inhibitory neuron as well. Inhibitory synapses also had identical weights and were set such that a spike of the inhibitory neuron reliably suppressed firing in the excitatory sub-network for typically a few milliseconds after the firing of an excitatory neuron (“winner-takes-all” mechanism).

The membrane potentials of all neurons followed a low-pass dynamics with time-constant τ_*m*_ = 10 ms and a reset when they reached firing threshold:

(1) $$\tau_{m}\frac{dV}{dt}=-V+S_{f}\text{ if }V\geq \theta \text{ then reset }V=0$$

Synaptic currents were described by variables, *S*_*f*_ and *S*_*r*_, and followed the dynamic equations:

(2) $$\begin{array}{l} \;\tau_{r}\frac{dS_{r}}{dt}=-S_{r}+I\\ \tau_{f}\frac{dS_{f}}{dt}=-S_{f}+S_{r} \end{array}$$

The simulations used forward Euler-integration with a time-step of *dt* = 0.1 ms.

The STDP rule used is a typical additive exponential STDP update rule. Pre- and post-synaptic spike-pairs evoked synaptic changes given by a function *f* of their temporal distance τ, see Equation (3). We used both, all-to-all and nearest-neighbor spike implementations (see Morrison et al., 2008), which differ only with respect to how many pre-synaptic spikes are considered: an all-to-all rule considers all pre-synaptic spikes whereas a nearest-neighbor implementation will only consider the pre-synaptic spike that is received closest to the time of post-synaptic activity. Equation (3) describes the STDP function used, where τ_*p*_ = τ_*d*_ = 20 ms. τ_*p*_ and τ_*d*_ were chosen similar to those observed experimentally (Levy and Steward, 1983; Bi and Poo, 1998; Wittenberg and Wang, 2006) where the strongest synaptic modifications occur within a window of ± 20 ms. Learning rates *A*_*p*_ and *A*_*d*_ for potentiation and depression were assigned using Equation (4). The maximum synaptic weight *W*^P^_max_ for Poisson afferents was assigned by Equation (5), where θ = 1, the cell's firing threshold, i.e. the difference in membrane potential required to go from resting to threshold, *r* is the average firing rate of afferents (64 Hz in most simulations), *N*_input_ is the number of afferents that carry the input pattern (herein 1000) and *A* = 20 is an additional constant that modulates the maximal strength of the synapses. This choice of *W*^P^_max_ allows for a reliable activation of neurons when the pattern is presented after training, but only few occasional output spikes due to random inputs. Note that the expression contains the integration step *dt*, because spikes are modeled as binary ones for one simulation time-step, such that shorter time-steps cause proportionally less post-synaptic activity; alternatively a spike-height of 1/*dt* could be used to make the spike impact independent of the integration step-size.

(3) $$\begin{array}{l} f(\tau )=A_{p}\;\times \text{ exp}\left(\frac{-\tau }{\tau_{p}}\right)\text{ if }\tau \geq 0\\ f(\tau )=A_{d}\;\times \text{ exp}\left(\frac{\tau }{\tau_{d}}\right)\text{ if }\tau <0 \end{array}$$

(4) $$\begin{array}{l} A_{p}=0.002\;\times \;W_{\text{max}}^{\text{P}}\\ A_{d}=-A_{p}\;\times \;\left(\frac{\tau_{p}}{\tau_{d}}\right)\;\times \;1.05 \end{array}$$

(5) $$W_{\text{max}}^{\text{P}}=\frac{\left[\frac{\theta }{\tau_{m}\;\times \;\langle r\rangle \;\times \;dt}\right]+A}{N_{\text{input}}}$$

Similar to previous synfire chain learning studies, we included weight normalization, Equation (6), so that the total excitatory lateral input to a neuron was no greater than *W*^L^_max_ = 50. The value for *W*^L^_max_ was found experimentally, cf., Figure 5.

(6) $$w_{i}=\{\begin{array}{ll} \frac{w_{i}}{\sum w_{i}}W_{\text{max}}^{\text{L}} & \text{if }\sum_{i=1}^{n}w_{i}>W_{\text{max}}^{\text{L}}\\ w_{i} & \text{if }\sum_{i=1}^{n}w_{i}\leq W_{\text{max}}^{\text{L}} \end{array}$$

Weights were initially set independent and identically distributed from uniform distributions: 0 < *w* ≤ *W*^P^_max_ and 0 < *w* ≤ *W*^L^_max_/N, for input and recurrent synapses, respectively.

Unless otherwise stated, training is run for a fixed duration of 200 s, at which time weights have practically converged (Figure 5). After training, the network performance is tested. We distinguish between “learning of a pattern” and “learning of a sequence.” This corresponds roughly to training of the feed-forward (ff) and the recurrent synapses, respectively.

A spatio-temporal pattern counts as learnt if a fixed set of neurons responds reliably to segments of it. Neurons are considered part of a trained chain only if they fire with a probability of at least 50% during repeated presentations of the input pattern in the test phase. The majority of neurons are much more reliable than 50% after training.

A sequence counts as learnt if the pattern is learnt (as described above), none but the first neuron fires with recurrent input switched off in response to the learned pattern, but they do fire in fixed order with recurrent synapses present. This implies that both the ff and the recurrent input into a neuron are needed to make it fire. A sequence counts as recognized during a presentation if all neurons of a learnt chain fire, especially the last one, which signals that a complete pattern has been seen by the network.

Due to the Poisson spikes on 1000 input lines and additional 10 Hz background spikes on all synapses, learning and retrieval are stochastic processes. Therefore, after training, neurons do not respond 100% reliable to the input pattern, but missing and spurious firings may occur caused by the random background input. This causes sequence recognition to fail and becomes more severe for longer sequences.

## 3. Results

### 3.1. Pattern learning for nearest-neighbor STDP

**Figure 2:**
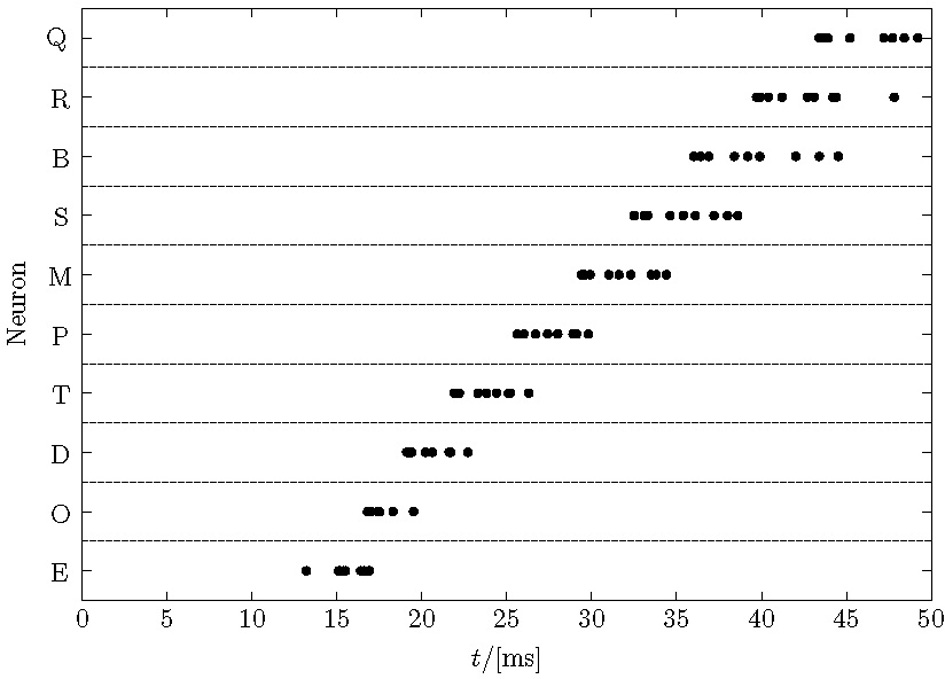
**Typical raster plot of output spikes in response to 10 presentations of a trained sequence.** After learning a repeated pattern of 50 ms duration, neurons in the network responded to unique segments of the pattern. The cumulative responses of 10 pattern presentations are shown, with jitter in the response times visible. Here, the order of firing is E→O→D→T→P→M→S→B→R→Q and each neuron responds in each trial but some spikes are printed on top of each other.

After learning a pattern with nearest-neighbor STDP, a set of neurons typically respond at different times during a pattern enforced by the winner-takes-all mechanism, see Figure 2 for an example. The figure shows the cumulative spikes of 10 neurons (Q–E) over time for 10 presentations of the trained pattern starting at time zero. Each line contains 10 accumulated firings (black dots; some printed on top of each other) reflecting proper sequence recognition for each individual pattern presentation.

Note that noise in the model is high—more than 75% of all spikes are noise spikes, because half of the input lines carry pure Poisson spike trains, the actual 50-ms training patterns are shorter than the periods of intermittent noise between their presentations, and there is an additional 10 Hz Poisson noise on each synapse. Therefore, even after training finished, the membrane potential fluctuations (not shown) are large such that the spike-times in Figure 2 display a considerable jitter. The distribution of response times becomes greater later in the pattern. The first neuron responds after about 15 ms reflecting the combined membrane and synaptic time-constants. These spikes are driven by the forward inputs only and quite reliable. Subsequent neurons fire in quicker succession driven by ff and recurrent input. Later spikes reveal a larger jitter because they rely on the ff input noise and the already jittered recurrent spike-times. The large noise limits the recognition rates that can be reached (see section 3.4).

**Figure 3:**
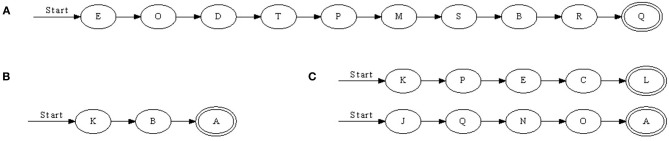
**Three chains learnt with nearest-neighbor STDP. (A)** Is a relatively long sequence consisting of 10 neurons whereas **(B)** and **(C)** are shorter. In **(C)**, two chains were learnt; when the pattern was presented, two sets of neurons responded in sequence. Double-line neurons are accepting neurons.

We found that with nearest-neighbor learning recurrent synapses between successive neurons were strengthened, leading to chains where each neuron in a sequence primed the next (Figure 3). Sometimes multiple chains would be learnt (Figure 3C), each responding to the same pattern. In such a case, all chains respond simultaneously to a pattern. This situation becomes more prominent when the number of neurons is increased. A 50 ms pattern requires only a relatively small number of neurons to be represented. A significantly larger number of neurons then allows for multiple representations.

### 3.2. Convergence of learning

**Figure 4:**
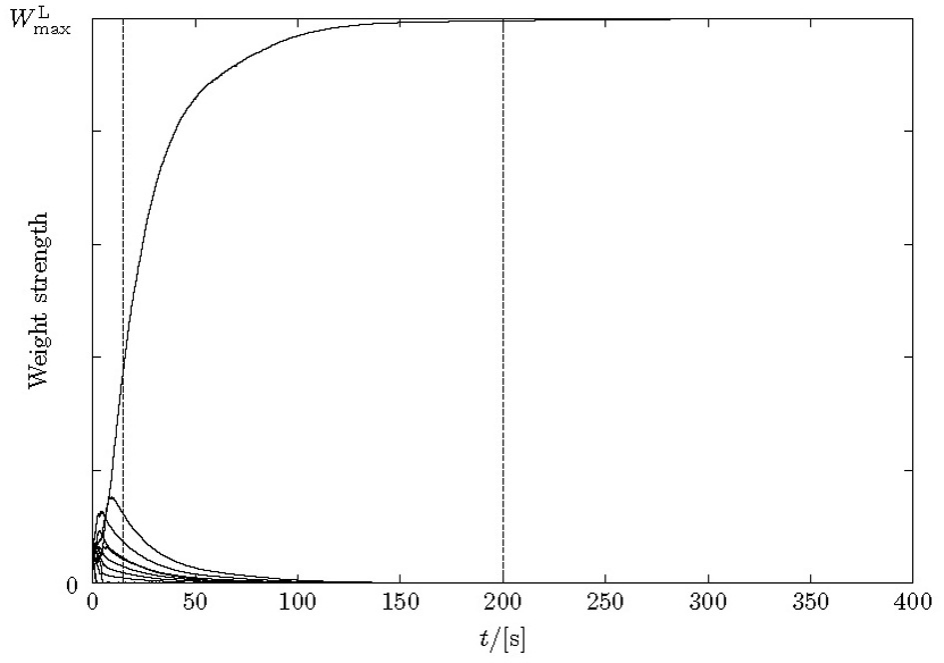
**Weight changes over time for lateral excitatory synapses during learning.** Only 10 synapses are depicted for simplicity. After 15 s the pattern is already learnt, although the weights do not fully stabilize until about 200 s.

Input to the model is stochastic. Even after long simulation times synaptic facilitation and depression events can happen by chance at individual synapses. Therefore, strictly speaking, weights never converge to deterministic limits but remain fluctuating even though with potentially small standard deviation. Whether, well-defined asymptotic weight distributions exist is likely, but given the analytically tough winner-takes-all dynamics those are difficult to predict. Beyond this general remark, Figure 4 provides some insight into the learning dynamics of the recurrent connections [aspects of training the ff synapses are studied in Masquelier et al. (2008), Humble et al. (2011) and not duplicated here].

Synapses start randomly initialized in a range [0, *W*^L^_max_/*N*] with *N* = 20. Because total depression exceeds total excitation in the STDP learning curve, pure random spike trains depress synapses when output neurons fire noise-driven. This happens during the first few seconds of the simulation (cf. Masquelier et al., 2008; Humble et al., 2011). The repeating pattern counteracts this weakening, leading to the development of specific ff synapses for segments of the input in some output neurons. Subsequently recurrent synapses develop that chain output neurons together according to the temporal structure in the input.

The amount of time required for a network to learn a pattern according to our 50% criterion is a few seconds (simulated time) given the parameters in section 2. Sequences were learned in between 10 and 20 s, or at most 100 stimulus presentations. The mean length of learnt sequences was five neurons. The learning of weights continues after the sequence is learnt (15 s in Figure 4); weights stabilize much later (200 s in Figure 4). After learning, lateral weights had values close to zero or the maximally possible value.

### 3.3. Effect of lateral weight bounds

**Figure 5:**
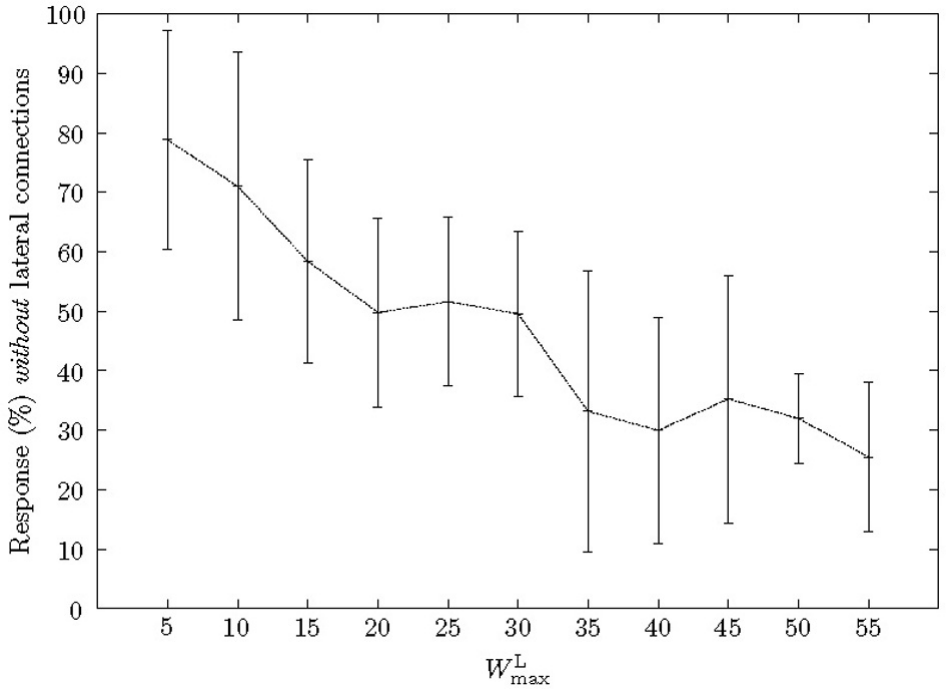
**Effect of *W*^L^_max_ on a neuron's dependence on previous neurons.** As the maximum strength of lateral connections, *W*^L^_max_, was increased, neurons became more dependent on the input from antecedent neurons. Due to STDP in the lateral connections the number of neurons responding without strengthened lateral connections decreased accordingly.

To analyse the effect of *W*^L^_max_ on learning we varied the parameter and measured the percentage of neurons, which had learnt a pattern that responded with severed lateral connections (Figure 5). When *W*^L^_max_ was increased the percentage of neurons that responded within the pattern *without* lateral connections decreased. In other words, as *W*^L^_max_ increased a neuron depended more on precedent neurons. Specifically, when *W*^L^_max_ was low (*W*^L^_max_ = 5), 80% of neurons responded without lateral synapses, however, this decreased to ≈25% with stronger lateral synapses (*W*^L^_max_ = 55). Furthermore, very strong lateral synapses (*W*^L^_max_ ≥ 60) interfered with the common Poisson input as they had a relatively strong efficacy toward a neuron's firing and therefore impeded with the learning.

When *W*^L^_max_ = 50, the start neuron was routinely the only neuron which responded without lateral connections. Furthermore, we found that an accepting neuron only responded when a pattern was presented in full. For example, if a pattern was reversed a chain's last neuron (and usually many more) would not respond. This effect was independent of the number of neurons and only depended on the absolute value of *W*^L^_max_.

### 3.4. Impact of other model parameters on learning and retrieval

Over 30 trials the success rate for learning patterns was 100% meaning that a reliable set of neurons practically always developed that reflects segments of the input patterns. However, this does not imply that individual neurons respond 100% reliably to their respective input segments. Given the large noise in the system, even after long learning periods a residual probability for missing or spurious firings remains. For sequence retrieval the probability of errors accumulates, which leads to a success rate for learning sequences of 62% given the parameters used.

The noise level can be adapted by changing the background firing rate or the number of background inputs. This, however, is possible only in limits due to the special nature of the model: The input noise is integrated over time by the membranes where it forms temporally correlated Gaussian noise (asymptotically for many inputs). Because all neurons receive precisely the same learning pattern reducing the standard deviation of the noise would make the membrane potentials of all neurons more and more similar. A certain noise level is needed to break this symmetry and assign different neurons to different segments of the input pattern (with the additional help of the winner-takes-all mechanism). On the other hand, increasing the standard deviation of the noise also increases the error probabilities for spurious and missing spikes during retrieval. This can in turn lower recognition rates.

Changing the pattern duration has previously been studied by Masquelier et al. (2008). Masquelier and colleagues found that as pattern duration increased the performance of pattern learning dropped to 59% with 100 ms and further to 46% with 150 ms; this effect would impede the learning of chains in this study and we therefore kept pattern duration at 50 ms. The decrease is due to the STDP rule which depresses synapses if they see Poissonian input and output spikes, because overall LTD dominates LTP in the learning rule. A pattern embedded in noise must therefore appear sufficiently frequently in order to facilitate synapses. As patterns get longer their presentation frequency decreases, which impairs learning.

The number of competing neurons (*N*) could be changed, but increasing the number too much interferes with learning for similar reasons as longer patterns: During the initial learning phase more neurons compete for a segment to learn, such that they respond less often because competitors do. Thereby the chance for facilitation becomes reduced and depression dominates.

Overall the parameter dependences discussed in this section suggest that the original model from Masquelier et al. (2008, 2009) can be improved for optimal sequence learning. We come back to this point in the discussion.

### 3.5. Results for all-to-all STDP

**Figure 6:**
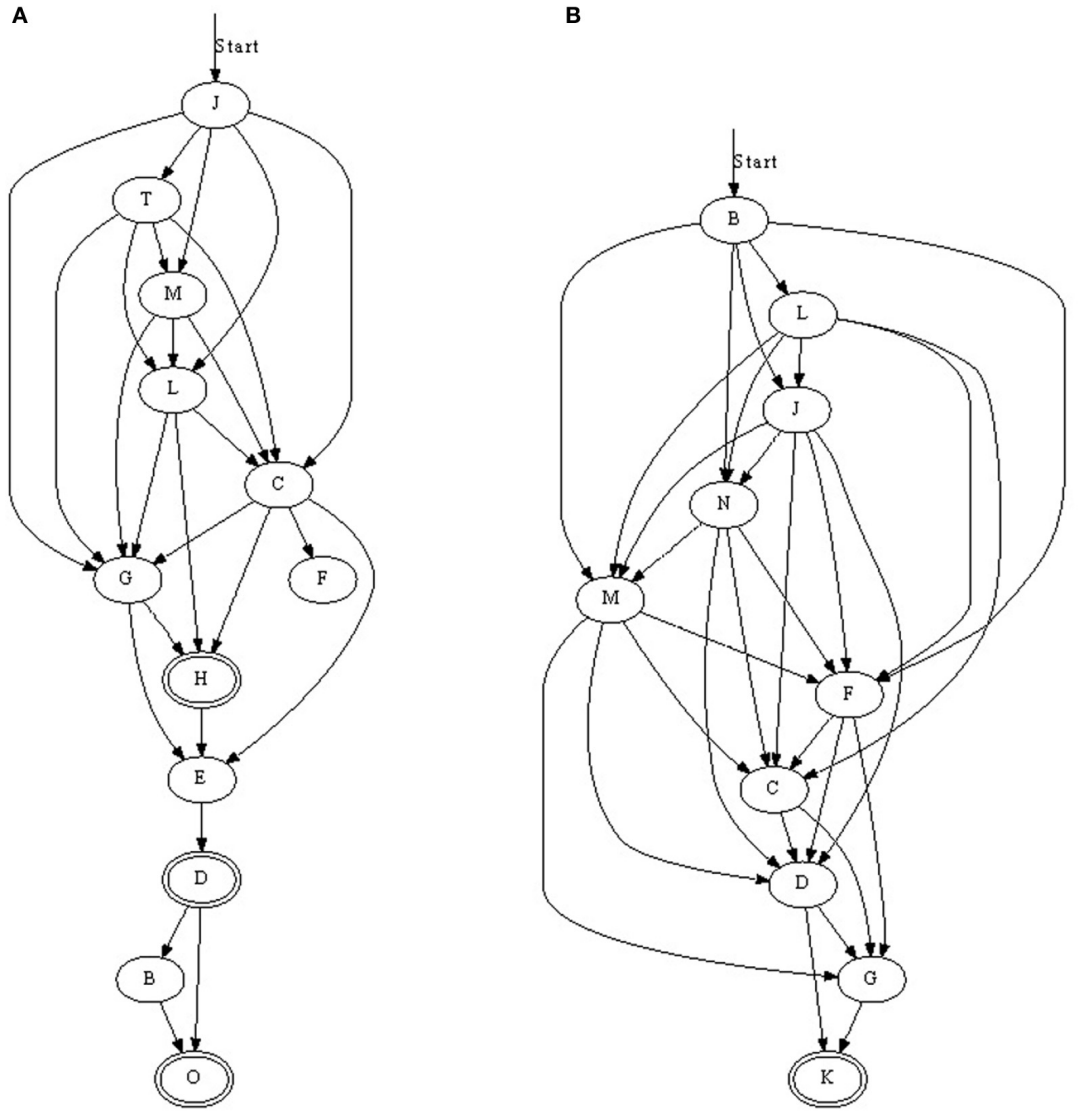
**(A,B) Two synfire chains learnt with all-to-all STDP.** With all-to-all STDP, more synapses were strengthened resulting in some connections straddling subsequent neurons. For example, in **(A)** neuron C not only receives input from the previous neuron, L, but also M and T. Double-line neurons are accepting neurons.

When all-to-all STDP is used instead of nearest-neighbor STDP more than one outbound/inbound connection per neuron was strengthened (Figure 6). For example, if A→B→C→D was learnt with nearest-neighbor spike STDP, all-to-all STDP would add A→C and B→D, and possibly A→D, if the temporal window of the learning rule and the spike-times allowed for this. As with the last chain in Figure 3, the connections do not always form in one single ordered sequence, but more complex situations are possible. For example, three accepting neurons are present Figure 6A: neuron O will respond if the entire pattern is presented and neurons D and H will respond when the majority of the pattern is presented. However, these cases were rare and the majority of chains had one accepting neuron. The presence of more than one accepting state was due to some neurons responding only just above 50% accuracy. In Figure 6A for example, neurons D and O would not respond 100% of the time and in the cases where they did not respond neuron H was the final neuron.

### 3.6. Training more than one pattern at a time

**Figure 7:**
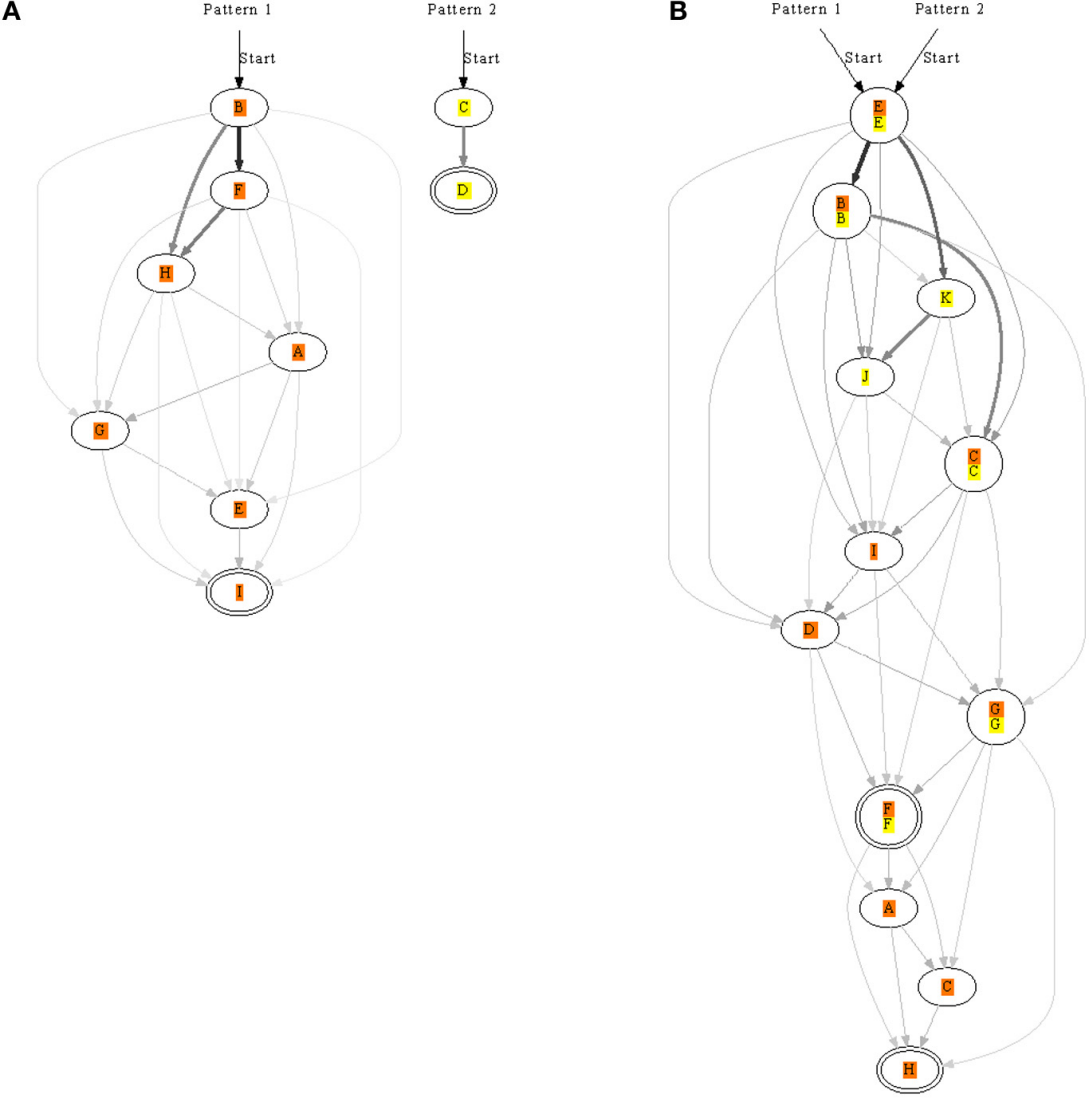
**Synfire chains learnt for two patterns, orange and yellow, presented separately to the same network.** In **(A)**, each pattern is recognized by a different set of neurons. In contrast, the patterns in **(B)** are both recognized with two overlapping pools of neurons. The strength of synapses is represented by edge color and thickness; for example, a thick black edge represents a strong synapse. Double-line neurons are accepting neurons.

Next we looked at presenting two patterns to see if more elaborate chains could form. The patterns were randomly presented but always non-overlapping. We usually found that both patterns were learnt (Figure 7), although, sometimes only one of the patterns was learnt. In addition to a neuron learning one pattern, some neurons learnt both patterns and took part in both chains. Networks therefore were often complicated with multiple pathways.

**Figure 8:**
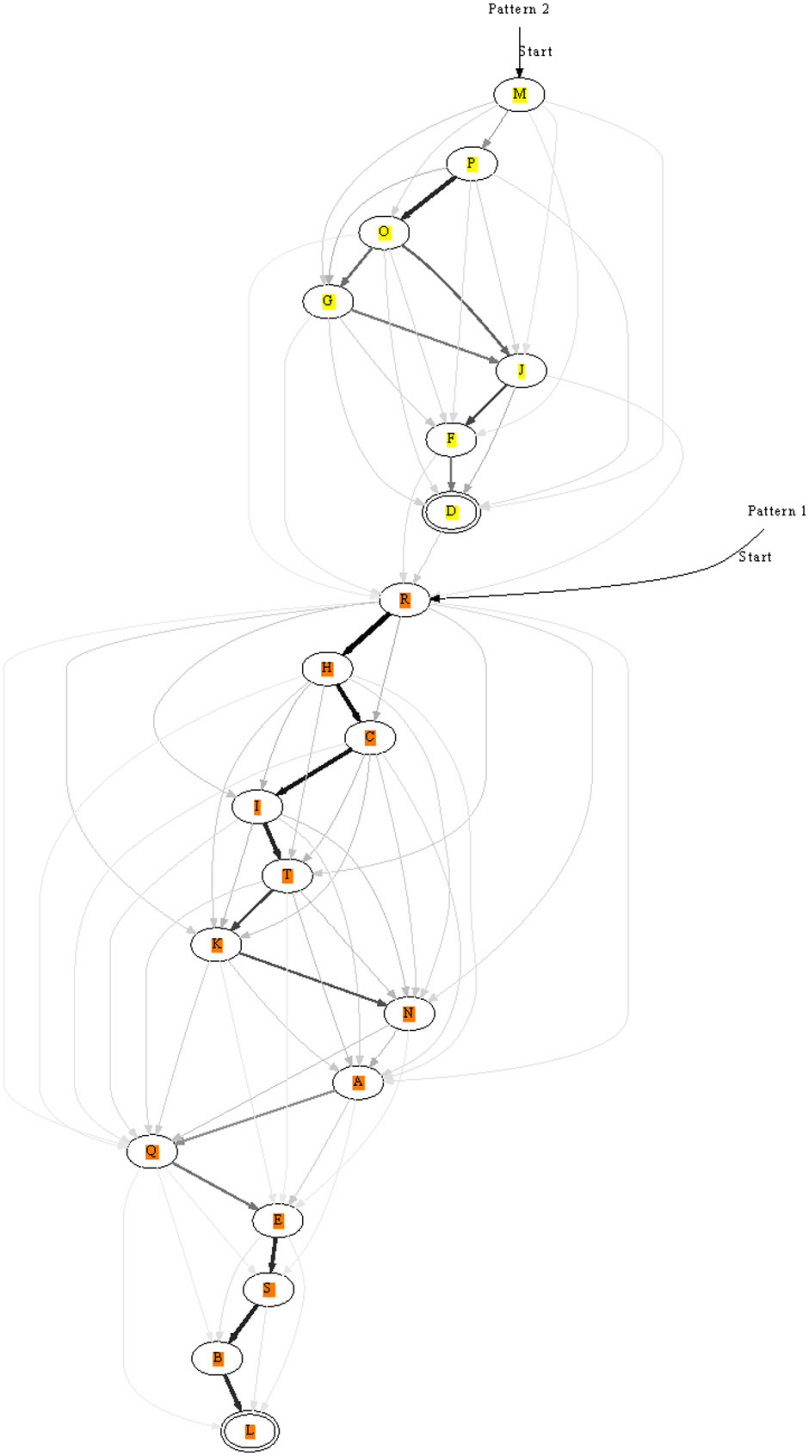
**A long chain formed from two shorter ones each recognizing a different pattern.** After learning the two patterns separately, they were presented in succession: pattern two → pattern one. The end of pattern two connected to the beginning of pattern one. Double-line neurons are accepting neurons.

As several lateral connections now drove each neuron we analysed the strength of these synapses: generally the more direct the connection the stronger the synapse. In a chain A→B→C for example, the synapse between A and B would usually be stronger than that between A and C. Synaptic strengths are indicated in Figures 7, 8 to illustrate the fact.

### 3.7. Concatenating chains by training

In the previous sections we learnt only short patterns. The length of patterns can in principle be increased, but we tested an alternative option: the chaining of already learnt segments. For this purpose two patterns were stored as before, but in a second phase the two patterns were repeatedly presented consecutively. This allowed two chains to connect, see Figure 8 for an example. If before learning two patterns were recognized by two unique chains, then the end of one would be linked to the beginning of the other. Still, recognition of the individual sub-chains remained possible using appropriate entry and exit nodes along the combined chain.

This mechanism in principle allows to train sequences hierarchically and potentially also to form more complex graph-like structures from simpler elements.

### 3.8. Notes on memory capacities

It may seem that using 20 neurons to store two patterns makes inefficient use of resources. This, however, deserves further consideration. Observe that two types of connections in the model store information, the ff synapses and the recurrent connections in the output layer.

Each trained neuron in the output layer responds specifically to a short segment in the input of length roughly similar to the duration of post-synaptic potentials; this is the time over which synaptic integration efficiently takes place in the ff synapses, and it is of the order of 10 ms. Segments coded by subsequent output neurons overlap to some degree (see Figure 2 where distinct output neurons fire about every 5 ms). The output network therefore basically uses a one-out-of-*N* code to represent short segments of the full spatio-temporal input pattern. If two spatio-temporal patterns each of 50 ms duration are stored in 20 neurons this gets quite close to the maximally possible number of segments. The pattern capacity of a one-out-of-*N* code is α: = *P*/*N* = 1 or, informally, as many segments *P* can be represented as there are neurons, *N*. The recurrent connections chain these segments in the order of their occurrence.

It is known from theories of associative memories that sparse codes can have a much higher pattern capacity than dense codes and one-out-of-*N* codes. For example, the memory capacity of the Hopfield network with dense memory patterns (that is, a probability of *p* = 1/2 ones and minus ones each) is α = P/N ≈ 0.14, whereas for sparse codes with *p* percent ones (and zeros else) the capacity is of the order of α ~ −1/(*p* log *p*). As *p* becomes small this number can become very much bigger than 1 (as compared to a constant of 0.14 in the Hopfield net). Both results hold asymptotically in very large networks, but approximately also in smaller networks (Palm, 1991).

This suggests that a *k*-winner-takes-all mechanism which selects *k* output neurons sparsely instead of only one, could be much more efficient regarding pattern storage capacities in the recurrent connections of the output layer. Unfortunately, it is not obvious how to design a neurally plausible mechanism that selects *k* neurons reliably and randomly during learning. Bienenstock noticed that the number of sets of *k* out of *N* neurons grows quickly in the sparse region, which makes a threshold mechanism to reach a controlled level of activity *k* and selects the same *k* neurons under the same input conditions unreliable (Bienenstock, 1995). Also, in a case where learning is ongoing, some neurons will have already strengthened synapses relative to others which biases them toward higher firing probabilities and therefore a higher rate to be selected again. This is not only unwanted because it reduces memory capacities (high capacities assume independent neurons in patterns) but also in practice often leads to a core of neurons developing many mutual synapses, whereas other neurons never learn. The problem of designing a reliable *k*-winner-takes-all mechanism that avoids these problems has to be left to future work.

How much information is contained in the forward synapses of the network is also quite close to a theoretical limit given a one-out-of-*N* code in the output layer. Note that given firing rates of roughly 50 Hz (or 1 spike per 20 ms) in the spatio-temporal patterns to learn, a neuron fires with probability 1/2 in a segment of about 10 ms duration. Because binary patterns with independent 0/1-entries and *p* = 1/2 contain maximally possible information the learnt segments per output neuron in our model have a high information content close to maximum. This, of course is only an estimate; a more elaborate theory would have to take threshold and noise levels into account as well as jitter in spike-times. This is out of the scope of the current work. Note also that again, sparse patterns in the input and output layers may potentially increase the pattern capacity or total information stored in the forward synapses.

### 3.9. Invariance against stimulus speed

Earlier work implies that it is possible to reach a certain amount of invariance in pattern recognition if the speed of the input pattern is changed. This may require an adjustment of the firing thresholds (Wennekers and Palm, 1996; Wennekers, 2000).

**Figure 9:**
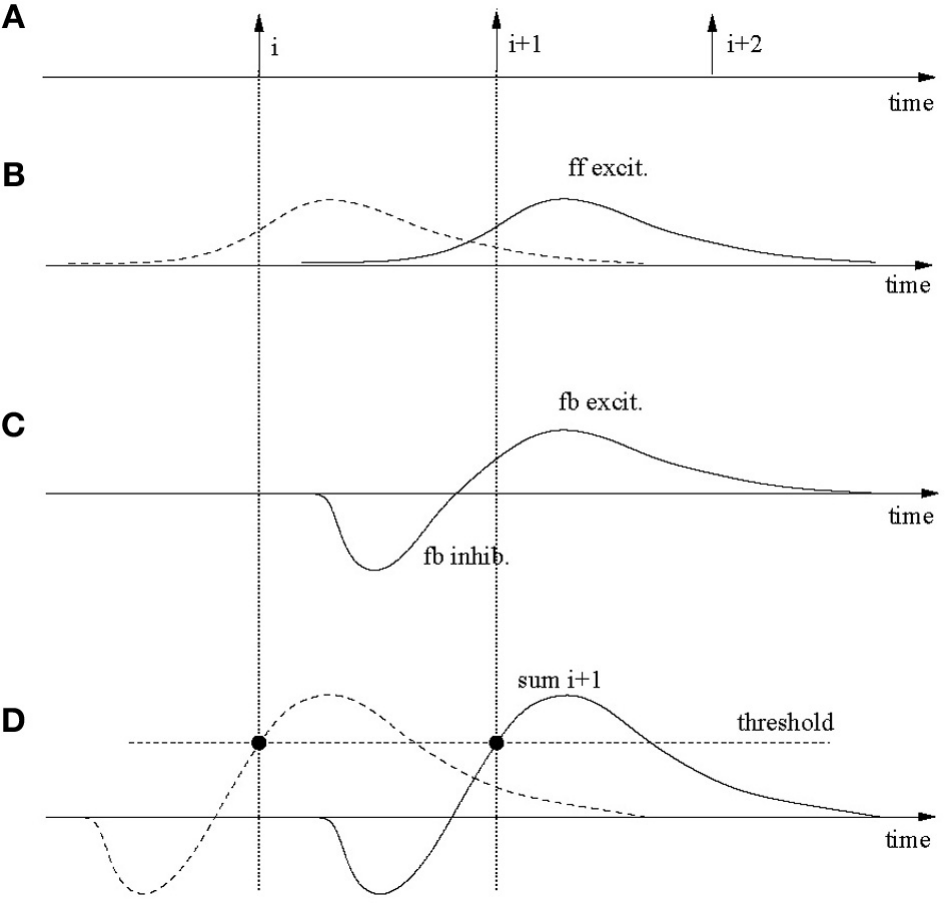
**Robustness against input speed variations. (A)** Spikes in three consecutively firing output neurons. These spikes are partly caused by specific feed-forward input **(B)** and the recurrent feedback connections **(C)**. The dashed post-synaptic potential in **(B)** contributes to the firing of neuron *i*, the solid line to *i* + 1. **(C)** Excitatory-inhibitory post-synaptic signals on neuron *i* + 1 due to specific excitatory feedback (fb) from neuron *i* and the global inhibition also triggered by the firing of neuron *i*. **(D)** The feed-forward and feedback signals superimpose on the output neurons and cause spikes where firing thresholds are crossed. These times must be the same as in **(A)** for consistency. If an input gets (slightly) faster than during training signals in **(B)** get compressed in time but larger in amplitude, which (slightly) moves the threshold crossing. By continuity, threshold variations can be used to speed up (or slow down) the recognition speed in the output network.

Figure 9 demonstrates the main mechanisms. Sub-plot A shows spike-times of three consecutive neurons in the output layer, which for simplicity are assumed to fire at regular intervals. Each firing requires ff input from the spatio-temporal driving pattern. This is indicated in sub-plot B where the solid line applies to spike *i* + 1 and the dashed line to spike *i*. These post-synaptic potentials are caused by brief segments before the spike that utilize trained, pattern-specific synapses. After a spike in neuron *i*, the inhibitory loop is triggered as well as an excitatory post-synaptic potential is elicited on neuron *i* + 1. The superposition of both is exhibited in sub-plot C. Sub-plot D shows the sums of the feedback (fb) inhibition and ff and fb excitation for neurons *i* (dashed) and *i* + 1 (solid). The neurons fire where the sum reaches the firing threshold (solid circles). The spikes-times in A and the threshold crossing in D coincide for reasons of self-consistence.

Now assume that the input is slightly slower (or faster). In that case the curves in B will be slightly stretched and lower (or compressed and higher), whereas those in C stay the same. This will furthermore lead to slight changes in the summed curves in D, however, as long as the changes are small, the curves and changes are continuous. Therefore, the threshold can be slightly adapted to have neuron *i* + 1 fire at a slower (or higher) speed as required by the input. Simulations actually show that the system tolerates a certain amount of noise in the input, which implies that the system is stable; for small enough speed changes a threshold adaptation is not required.

Even though the above analysis applies to the model in this paper, the speed changes that can actually be tolerated are quite small for two reasons. At first, the rise-times of the post-synaptic membrane potentials are small only (a few milliseconds). Because the threshold crossing causing the firing of neurons must fall on the rising edge, the maximum amount of speed tolerance is thereby limited. A second limiting factor is the large input noise for the parameters used in accordance with previous works. The input noise causes large membrane fluctuations which have an impact on the stability of wave propagation. On one hand, in order to allow any reliable retrieval, the threshold should be more than one standard deviation of the noise below the maximum in the summed potentials in Figure 9; otherwise a neuron in the chain may miss firing by a significant probability which would terminate the chain. On the other end, the threshold must also be sufficiently bigger than both the ff and fb excitation, again, by more than a standard deviation of the noise. Otherwise neurons may easily get excited randomly. It turns out that given the parameters used in the simulations the noise is so large that this leaves only a relatively small region for proper threshold settings. This is, for example, also indicated by Figure 2 where even with a properly set threshold, spike jitter of output neurons in repeated simulations are around a few milliseconds, much comparable with the rise-times of the post-synaptic potentials.

Finally, let us briefly mention that the above picture is not entirely complete. It leaves out a (constant) potential offset due to the Poisson inputs, membrane potential resets of the leaky-integrate-and-fire neurons after firing, and the fact that the recurrent inhibition acts globally. These effects have an impact on the precise shape of the membrane potential and firing times but do not change the main arguments qualitatively.

## 4. Discussion

It has previously been found that several neurons can learn to recognize different sections of a repeated spatio-temporal pattern (Masquelier et al., 2009). We studied whether it was possible to join these consecutively firing neurons into an ordered chain that recognizes a spatio-temporal input pattern. Using both nearest-neighbor and all-to-all STDP it was possible to form chains using a simple network of excitatory neurons laterally connected and one inhibitory neuron providing winner-takes-all competition.

Previous attempts to learn synfire chains (Bienenstock, 1991; Prugel-Bennett and Hertz, 1996) have set-up random networks and some have excited neurons synchronously to strengthen connections. Our approach was different: we had a simple network that was driven mainly by a repeated spatio-temporal pattern where all excitatory connections were plastic for the duration of a simulation. Furthermore, a major limitation of previous studies was that cyclical chains would form. These closed loops interfered with the learning of long chains as once activity enters a loop it doesn't exit. None of our chains had cycles because the network was driven by a specific stimulus instead of random activity as with previous studies. This stimulus driven set-up deterred the formation of loops because the repeated patterns were separated by random activity that was not learnt.

An important result of having the lateral connections plastic throughout the learning process was that subsequent neurons in a chain not only recognized when a pattern segment was presented, but crucially only did so if the previous neuron had fired as well. The chains are therefore effectively simple finite automata that recognize linear sequences. However, the lateral connections only had this effect if they were allowed to strengthen enough (Figure 5). When a neuron receives input through a lateral connection from a precedent neuron, this increases the membrane potential and primes the neuron to fire on appropriate input from the Poisson inputs.

We tested the possibility to learn longer sequences hierarchically from shorter segments representing two subsequent patterns. The longer chains that formed, included at least one accepting neuron that only responded if a learnt pattern was presented in full correctly. For example, if the pattern was reversed or split in half the accepting neuron would not respond. This multiple order dependence on previous neurons allowed for multiple paths through a network to be learnt.

Given that due to the random inputs firing was to some degree stochastic, the structures learned using nearest-neighbor learning may be considered to be similar to first-order Markov chains. To extend this interpretation of learnt sequences as Markov chains, we found it was possible to learn higher order chains with all-to-all STDP. These produced neurons which not only relied on a previous neuron's response and the appropriate pattern input, but to a certain extent on the temporal pattern of activity across several neurons and time-steps. This dependence on previous neurons reinforced the concept of an accepting neuron only responding if a pattern had been presented in full.

Results shown confirm a previous finding that the level of background activity present during and after learning needs to be similar for proper pattern recognition (Humble et al., 2011). Specifically, if the level of background activity increases after learning, a neuron may respond earlier, and if the activity is decreased, a neuron may no longer respond at all. These effects can impair the recognition of stimuli. However, it should also be noted, that the level of background activity allows the control of the speed of activation patterns in synfire chains (Wennekers and Palm, 1996; Wennekers, 2000). Such a mechanism can be used to gain a certain invariance against the replay speed of learned patterns. However, given the high noise level used in the present work [for better comparison with previous models of Masquelier et al. (2008)] the controllable range is only small.

The high noise level also implies relatively low sequence recognition rates. The noise level, cannot be simply reduced because as discussed in section 3.4 at low noise levels membrane potentials become more and more similar because all neurons receive the same input. In this case the winner-takes-all mechanism has difficulties to reliably select only one neuron. Noise is needed to spread the membrane potential distribution and randomly select output neurons during learning. If neurons would not all see the same input, the noise level could likely be lowered and the recognition rates increased. This may improve the performance of the model.

Another drawback of the model is the relatively simple standard STDP learning rule. As mentioned in section 3.4 and already observed by Masquelier et al. (2008) the rule limits the maximum length of trainable patterns. In response to Poisson spikes synapses depress such that a certain minimum frequency for training patterns to repeat is required to facilitate synapses. The inverse of this frequency limits the maximum duration of trainable patters. We are currently testing other learning rules to overcome this problem.

The weight normalization used in our study may be seen a limitation. However, previous studies have used similar forms of weight normalization; it seems to be crucial for the formation of synfire chains (Bienenstock, 1991; Prugel-Bennett and Hertz, 1996). Our implementation of weight normalization is not local and would therefore require some signaling in biological neurons. Whether such signaling or weight normalization is present within neurons is not known. Furthermore, the calculations for the maximal afferent weight for both input and lateral synapses (*W*^P^_max_ and *W*^L^_max_) would also require some, as yet, unknown signaling. However this would ideally be an “inbuilt” property of neurons.

Another limitation of our network is its small pattern capacity. Neurons can typically not be trained to participate in more than two patterns. This limitation is mainly due to the non-sparse input with 1000 inputs representing the signal and another 1000 carrying Poisson noise. The study of sparse input and output patterns suggested in section 3.8 remains an interesting but open problem. Sparse codes are known to drastically increase pattern capacities in associative memories (Palm, 1991). In the present context they may similarly help with (1) overcoming the need to have about 20 neurons to recognize “only” 100 ms of input and (2) increasing the number of different patterns stored within a network.

Experimental evidence is accumulating, that cortical activity is often sparse and precisely timed in response to repeating stimuli. (Wolfe et al., 2010) review evidence from a variety of species and cortical areas showing that neurons can respond highly selectively in response to brief periods of repeating inputs. The selectivity and sparseness is controlled by inhibitory networks of neurons which also chop the excitatory responses into bursts in the beta frequency range. Our model acts in a similar way causing sequences of neurons to fire in response to specific segments in the inputs. The firing activity in the model is rhythmic due to the winner-takes-all inhibition which causes sparseness, i.e., the firing of only a single neuron per recognized segment. A *k*-winner-takes-all mechanism may reflect the biological situation even closer. Our model, however, has the additional feature that on top of neurons that recognize single short segments of the input, we also utilize recurrent excitatory connections to encode longer patterns. It may be worthwhile to explore this possibility in experiments by stimuli that either support the propagation of activity (the original pattern) or not (the reverse pattern or scrambled segments). The possibility to stitch patterns together can also be tested experimentally. In the present work we have only shown this for two patterns (due to limitations of the learning rule) but in principle chains could be combined into more complex patterns that may underlie cortical computations.

To summarize, it is interesting to see that a simple network of laterally connected excitatory neurons can self-organize into spatio-temporal pattern recognizers. Simple Markov-like synfire chains were learnt with nearest-neighbor STDP and more complicated multiple order chains with all-to-all STDP. Furthermore chains recognizing different patterns could be joined to represent longer, potentially nested patterns. Even though some limitations must be overcome, the latter offers the possibility to learn graph-like transition patterns with stronger computational capabilities than linear sequences, for example, as suggested by Wennekers (2006) or Izhikevich (2009).

### Conflict of interest statement

The authors declare that the research was conducted in the absence of any commercial or financial relationships that could be construed as a potential conflict of interest.
